# Identification of Interphotoreceptor retinoid-binding protein in the Schisis cavity fluid of a patient with congenital X-linked Retinoschisis

**DOI:** 10.1186/s12886-021-02234-5

**Published:** 2022-01-06

**Authors:** Fukutaro Mano, Koji Sugioka, Kazuki Kuniyoshi, Hiroyuki Kondo, Shunji Kusaka

**Affiliations:** 1 Department of Ophthalmology, Kindai University Faculty of Medicine, Osakasayama, Japan; 2 Department of Ophthalmology, Kindai University Nara Hospital, 1248-1 Otodacho, Ikoma City, Nara, 630-0293 Japan; 3 Department of Ophthalmology, University of Occupational and Environmental Health, Kitakyushu, Japan

**Keywords:** Congenital X-linked retinoschisis, Vitrectomy, Interphotoreceptor retinoid-binding protein, Proteomic analysis, Case report

## Abstract

**Background:**

This case report describes the surgical outcome in a patient with congenital X-linked retinoschisis (CXLRS) and the results of proteomic analysis of surgically extracted samples from both vitreous and intraschisis cavities by mass spectrometry.

**Case presentation:**

A 3-month-old boy presented with extensive retinoschisis involving macula and retinal periphery in both eyes. Genetic analysis confirmed retinoschisin 1 mutation (c.554C > T), and an electroretinogram showed significant reduction of b-wave and decreased cone and rod responses, which led to a diagnosis of CXLRS. By performing pars plana vitrectomy, including inner wall retinectomy, clear visual axes with stable retinal conditions and functional vision in both eyes were obtained during the 4 years of follow-up. Proteomic analysis of surgically retrieved fluid from the intraschisis cavity revealed a higher expression of interphotoreceptor retinoid-binding protein (IRBP) than that from the vitreous humor. However, both samples showed equal levels of albumin, transferrin, and pigment epithelium-derived factor.

**Conclusions:**

Cellular adhesive imperfection in CXLRS may cause IRBP diffusion from the interphotoreceptor matrix, resulting in the strong expression of IRBP in the intraschisis cavity. An impaired retinoid cycle caused by an absence of IRBP in the retina may potentially underlie the pathology of CXLRS.

**Supplementary Information:**

The online version contains supplementary material available at 10.1186/s12886-021-02234-5.

## Background

Congenital X-linked retinoschisis (CXLRS) is a retinal degenerative disease characterized by the splitting of retinal layers, disruption of photoreceptor-bipolar synapses, and photoreceptor degeneration [1]. The estimated prevalence of CXLRS ranges from 1 in 5000 to 1 in 20,000 [2]. Although patients with CXLRS have a broad spectrum of clinical phenotypes, a consistent feature of CXLRS is foveal schisis that is often described as a spoke-wheel pattern in a color fundus photograph [3]. Peripheral retinoschisis is found in > 50% of patients with CXLRS, most commonly in the inferotemporal quadrant [2]. The majority of retinoschisis cavities remain stable throughout life and yield preserved visual acuity. However, a select number of patients progress to macula involving retinoschisis, rhegmatogenous detachment, and combined tractional–rhegmatogenous detachments that require further intervention. Vitreoretinal surgical approaches remain complex, case-based, and require careful planning, depending on the configuration and location of the retinoschisis cavity [4].

The gene responsible for causing CXLRS has been identified as retinoschisin 1 (*RS1*), located on the distal short arm of the X chromosome (Xp22.1-p22.3), which encodes the protein retinoschisin [5, 6]. Retinoschisin is expressed on the surface of photoreceptors and bipolar cells and maintains the integrity of the retina [7–11]. Clinically, microstructural defects of the photoreceptor outer segment are observed frequently in patients with CXLRS, which is linked closely with poor visual acuity [12]. However, the exact mechanism between protein abnormality in retinoschisin and the consequent photoreceptor degeneration is currently unclear.

Mass spectrometry (MS) has emerged as a powerful tool for proteome-wide analysis and is one of the key technologies in molecular and cellular biology [13]. It is used to identify biomarkers and molecular changes associated with specific disease mechanisms. Herein, we report a case of a patient with CXLRS with extensive retinoschisis involving macula and retinal periphery in which pars plana vitrectomy successfully cleared the visual axis and gained functional visual acuity. The interphotoreceptor retinoid-binding protein (IRBP) was identified by MS in the intraschisis cavity of this patient with CXLRS.

## Case presentation

**Fig. 1:**
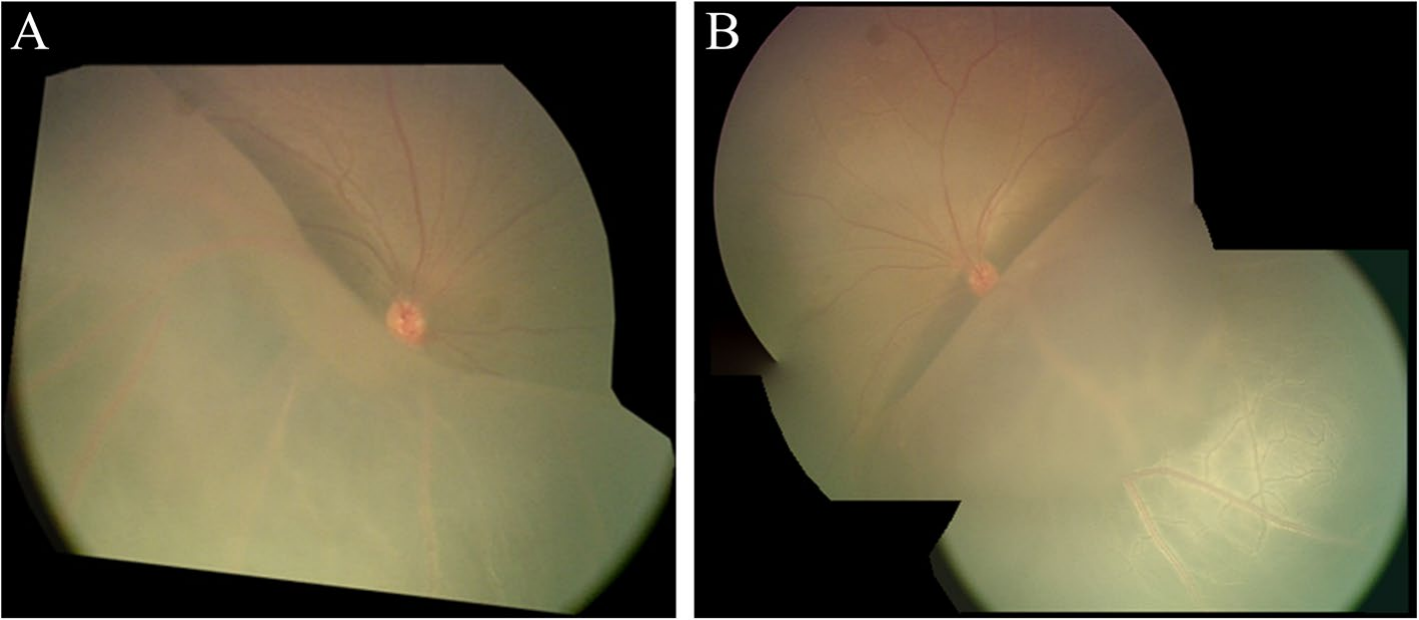
Peripheral retinoschisis overhanging the macula. **A** Bullous schisis cavity involves the macula in the right eye. **B** Retinoschisis cavity overhangs the macula from the inferotemporal quadrant in the left eye

**Fig. 2:**
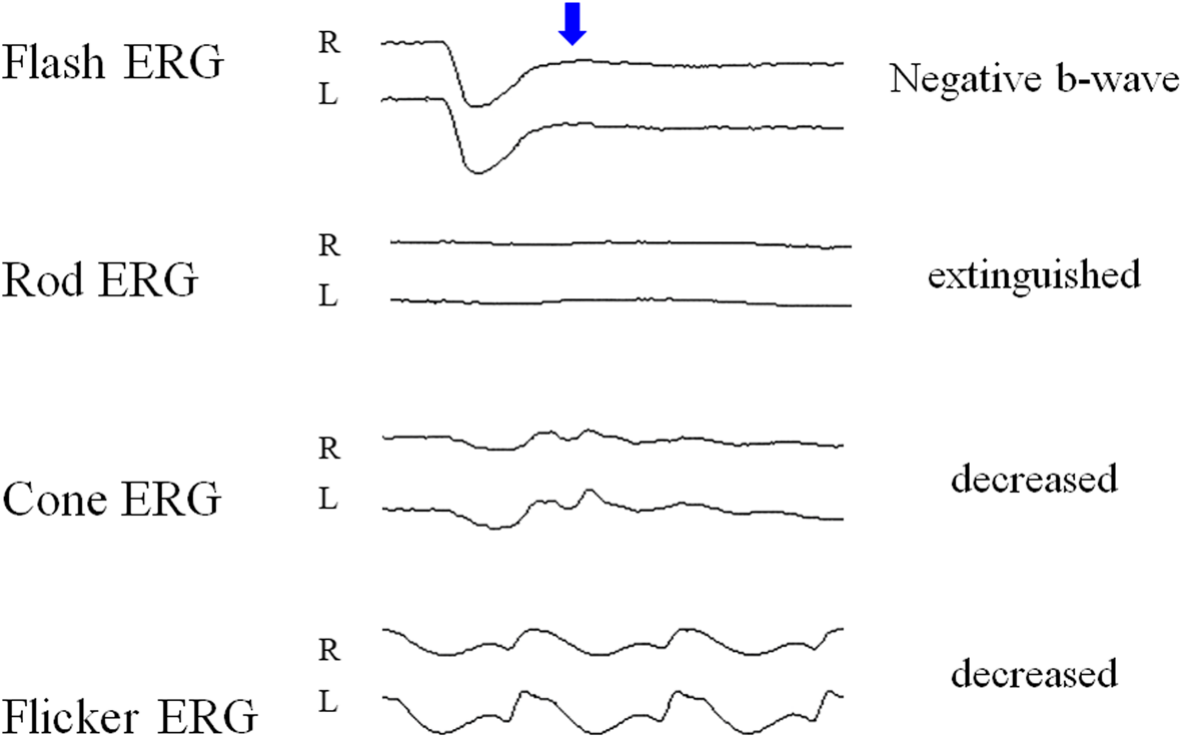
ERG findings in this patient. An electronegative b-wave was observed in the flash ERG (blue arrow). Decreased rod and cone responses and flicker ERG were also documented. ERG = electroretinogram

A 3-month-old boy presented with a poor fixation with occasional horizontal nystagmus detected by a local pediatrician. The boy was then referred to our department to evaluate the retinal detachment in both his eyes. The anterior segment was normal and the lens was clear. However, a bullous peripheral retinoschisis existed bilaterally overhanging the macula (Fig. 1 A and B), and peripheral retinoschisis was almost touching the lens. Genetic analysis confirmed the *RS1* mutation (c.554C > T), and an electroretinogram (ERG) demonstrated a significant reduction of b-wave and decreased cone and rod responses bilaterally (Fig. 2). The boy had no family history of visual impairment. Magnetic resonance imaging of the head revealed subdural effusion, but no other systemic complications were noted. Surgical intervention was proposed for this patient, and informed consent was obtained from the boy’s parents after discussing the benefits and risks of the procedure in detail.

**Fig. 3:**
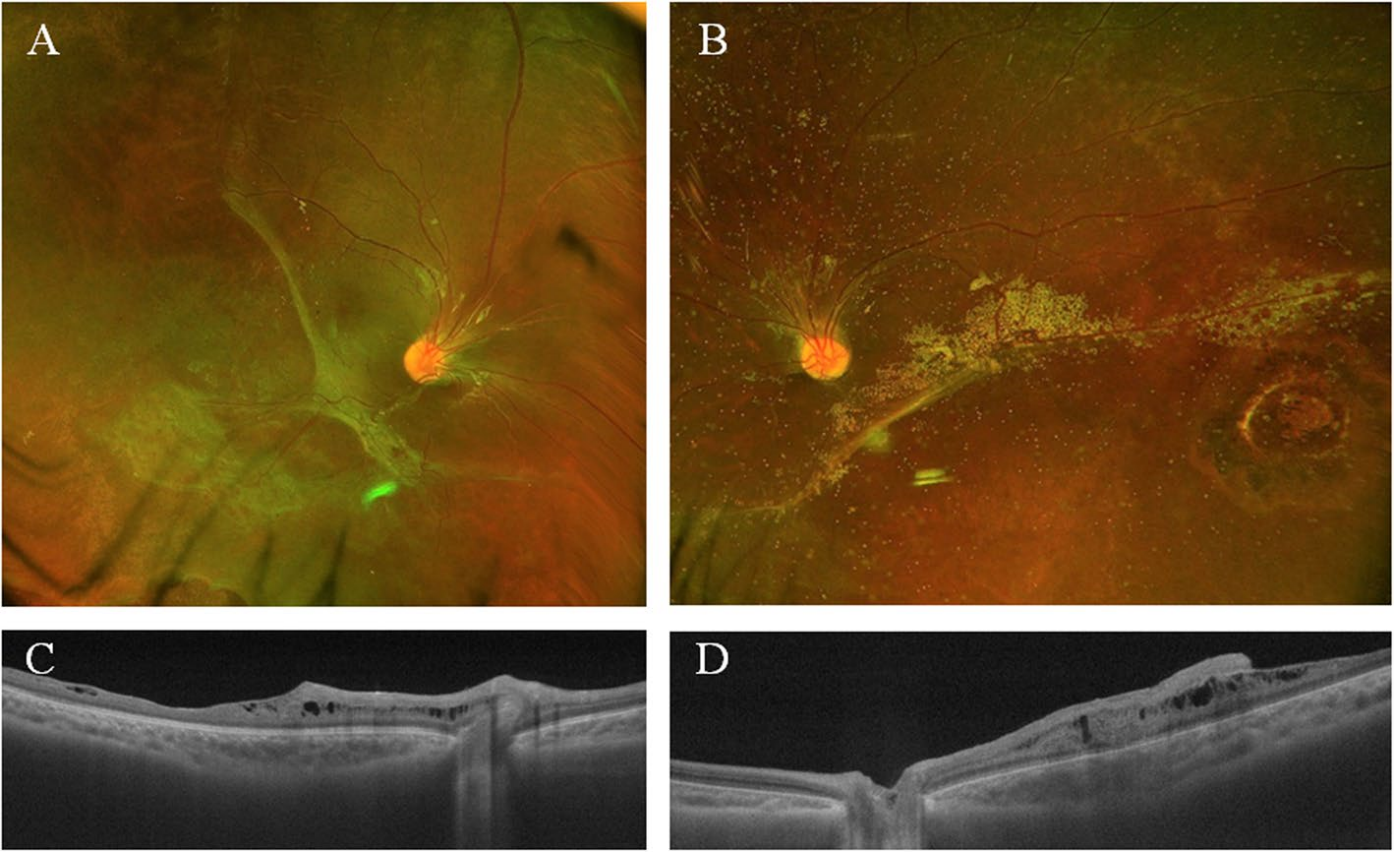
Fundus photograph and OCT 4 years after the surgery. **A** Color fundus photograph shows an attached retina in the right eye. Retinal vasculature was dragged nasally. **B** Color fundus photograph shows an attached retina in the left eye. Silicone oil particles were slightly observed. **C** OCT shows perifoveal cystic-like spaces within the outer plexiform layer. The inner retina was preserved, including the fovea. (D) OCT shows a large cystic-like space around the fovea. The inner retina includes the fovea. OCT = optical coherence tomography

The patient underwent lens-sparing vitrectomy in both eyes. After posterior hyaloid dissection, the fluid was drained through a small retinotomy site. Laser photocoagulation was performed around the retinotomy and causative inner retinal holes. Then, silicone oil tamponade was applied to the vitreous cavity. However, at 6 months after the initial surgery, a secondary cataract developed in his right eye. After cataract extraction and intraocular lens placement, we observed that emulsified silicone oil had migrated into the intraschisis cavity. Inner wall retinectomy was then enlarged and the silicone oil was retrieved (Video 1). His left eye developed a cataract over time, for which we performed cataract removal and silicone oil extraction 3 years after the initial surgery. A clear visual axis was obtained, and the retina in both eyes remained stable 4 years after the last surgery (Fig. 3). The postoperative optical coherence tomography showed cystic changes within the outer plexiform layer suggesting that the retinal splits seems to have occurred within that layer. The final visual acuity was 0.7 in the right eye and 0.3 in the left eye.

**Fig. 4:**
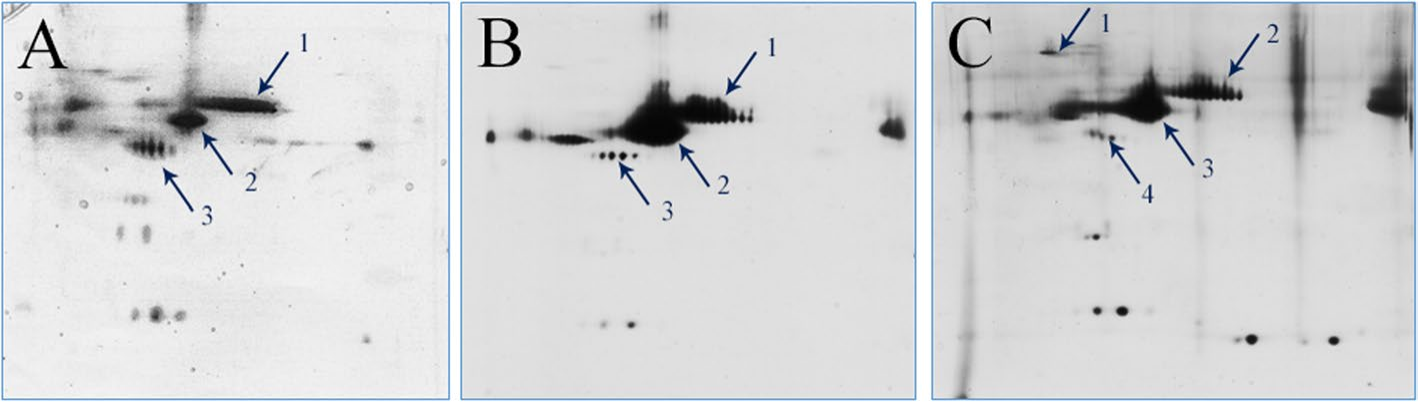
Two-dimensional gel electrophoresis of vitreous sample from (A) the congenital cataract, (B) the vitreous cavity of CXLRS, and (C) the intraschisis cavity of CXLRS. The proteins identified by mass spectrometry are (A) 1, transferrin; 2, albumin; 3, PEDF; (B) 1, transferrin; 2, albumin; 3, PEDF; (C) 1, IRBP; 2, transferrin; 3, albumin; 4, PEDF. CXLRS = congenital X-linked retinoschisis; PEDF = pigment epithelium-derived factor; IRBP = interphotoreceptor retinoid-binding protein

The samples extracted from both vitreous and intraschisis cavities at the initial surgery were separated by two-dimensional gel electrophoresis. The gels obtained were then stained with silver. The silver-stained proteins in the gel were excised, and the peptides were extracted as described previously [14]. A vitreous sample from a patient with a congenital cataract served as a control. Matrix Assisted Laser Desorption/Ionization-Time of Flight MS was performed using a Shimadzu Axima Performance (Kyoto, Japan), and the fragment ions were matched by searching against peptide mass fingerprints in the NCBI nr database using the Mascot search engine. Albumin, transferrin, and pigment epithelium-derived factor (PEDF) were found in all the samples. In particular, the band strongly expressed at the 140-kDa molecular weight position in the sample extracted from the intraschisis cavity was identified as IRBP by MS (Fig. 4).

## Discussion and conclusions

In this case of CXLRS, we report evidence that MS can be used to detect a higher expression of IRBP in the sample obtained from the schisis cavity than that in the vitreous sample and the congenital cataract sample. Severe b-wave impairment was observed in the ERG of our case, which could be consistent with a condition in the absence of IRBP.

The clinical expression of CXLRS is highly variable. In general, vitreoretinal surgery for a stable schisis cavity, which does not involve the macula, cannot be recommended in the majority of cases of CXLRS because of a high risk of long-term complications, including retinal detachment [15]. Nevertheless, vitreous surgery is often indicated for patients with the nonspontaneous resolution of vitreous hemorrhage or rhegmatogenous and/or tractional retinal detachment and an overhanging bullous schisis cavity threatening the macula [16]. In the present case, as the boy presented with a poor fixation with occasional horizontal nystagmus, believed to be caused by macula involving retinoschisis, we decided to perform pars plana vitrectomy. We successfully obtained a clear visual axis by performing pars plana vitrectomy and silicone oil tamponade in both the eyes. Inner wall excision was required in the right eye. Ideally, efforts should be made to preserve the inner wall retina, anticipating the future application of gene therapy in reapproximating the inner and outer retina [17–19].

Although the molecular role of retinoschisin within the retina has not been elucidated completely, it is believed to contribute to the development and maintenance of the cellular and synaptic organization of the retina [20, 21]. Retinoschisin is synthesized and secreted by photoreceptors, reaches the surface of other retinal cells, and mediates interactions or adhesion between photoreceptor, bipolar, and Müller cells to glue the retina together [9]. Joshi et al. identified tenascin-C as an extracellular matrix protein in the schisis cavity fluid of CXLRS [22]. As tenascin-C has been characterized as an antiadhesive molecule in various settings [23, 24], they concluded that the interaction between retinoschisin and tenascin may lead to extracellular matrix remodeling and the resultant antiadhesive environment within the schisis cavity [22].

In our case, the sample obtained from the intraschisis cavity of the right eye showed a higher expression of IRBP than that from the vitreous sample of the same eye and the vitreous sample from the other patient with congenital cataract. IRBP, also known as retinol-binding protein 3, was first discovered in soluble proteins extracted from the bovine interphotoreceptor matrix (IPM) [25]. IRBP is important for the development and maintenance of the visual cycle in humans. It mediates the extracellular diffusion of retinoids during the operation of the retinoid cycle, transporting all-trans-retinol and 11-cis-retinal between the photoreceptors and the RPE [26]. It has been reported that cone ERG responses in IRBP-knockout mice were reduced compared to those in control mice, although they had similar cone densities and opsin levels, indicating that IRBP is essential for normal cone function [27]. Similarly, in another study, rod-isolated response amplitudes were decreased by > 25%, and the cone response was reduced by > 50% in IRBP-knockout mice [28]. Moreover, mice lacking IRBP displayed severe early and progressive photoreceptor degeneration [29]. These findings related to dysfunctional IRBP share common clinical features with CXLRS [30, 31].

In addition, mice immunized with bovine IRBP were used as an experimental model of autoimmune uveitis [32], and anti-IRBP autoantibodies have been detected in patients with uveitis, retinitis pigmentosa, Coats disease, and age-related macular degeneration [33, 34]. Although anti-IRBP autoantibodies were not examined in our sample, autoimmunity toward IRBP might play a role in the inflammatory reaction within the schisis cavity of CXLRS.

Collectively, the susceptibility of cellular adhesion in CXLRS may cause IRBP diffusion from the IPM, resulting in the strong expression of IRBP in the intraschisis cavity. The absence of IRBP at the IPM may impair the retinoid cycle that could be clinically interpreted as a significant electronegative b-wave. Investigating the interaction between retinoschisin and IRBP may further elucidate the pathophysiology of CXLRS.

## Supplementary Information

**Additional file 1: Video 1.** Initial surgery for macula-involving congenital retinoschisis, and second surgery for cataract removal and inner wall retinectomy.

## Data Availability

All data generated or analyzed during this study are included in this published article.

## Abbreviations

CXLRS: Congenital X-linked retinoschisis
ERG: Electroretinogram
IRBP: Interphotoreceptor retinoid-binding protein
IPM: Interphotoreceptor matrix
MS: Mass spectrometry
RPE: Retinal pigment epithelium

## References

[1] Sikkink SK, Biswas S, Parry NR, Stanga PE, Trump D (2007). X-linked retinoschisis: an update. J Med Genet.

[2] George ND, Yates JR, Moore AT (1995). X linked retinoschisis. Br J Ophthalmol.

[3] Sieving PA, MacDonald IM, Hoang S, Adam MP, Ardinger HH, Pagon RA (1993). X-Linked Congenital Retinoschisis. GeneReviews(®). Seattle (WA): University of Washington, Seattle Copyright © 1993–2021, University of Washington, Seattle. GeneReviews is a registered trademark of the University of Washington, Seattle. All rights reserved.

[4] Rao P, Dedania VS, Drenser KA (2018). Congenital X-linked retinoschisis: an updated clinical review. Asia Pac J Ophthalmol (Phila).

[5] Sauer CG, Gehrig A, Warneke-Wittstock R, Marquardt A, Ewing CC, Gibson A (1997). Positional cloning of the gene associated with X-linked juvenile retinoschisis. Nat Genet.

[6] Kondo H, Oku K, Katagiri S, Hayashi T, Nakano T, Iwata A (2019). Novel mutations in the RS1 gene in Japanese patients with X-linked congenital retinoschisis. Hum Genome Var.

[7] Gieser EP, Falls HF (1961). Hereditary retinoschisis. Am J Ophthalmol.

[8] Wu G, Cotlier E, Brodie S (1985). A carrier state of X-linked juvenile retinoschisis. Ophthalmic Paediatr Genet.

[9] Reid SN, Yamashita C, Farber DB (2003). Retinoschisin, a photoreceptor-secreted protein, and its interaction with bipolar and muller cells. J Neurosci.

[10] Takada Y, Fariss RN, Tanikawa A, Zeng Y, Carper D, Bush R (2004). A retinal neuronal developmental wave of retinoschisin expression begins in ganglion cells during layer formation. Invest Ophthalmol Vis Sci.

[11] Molday RS, Kellner U, Weber BH (2012). X-linked juvenile retinoschisis: clinical diagnosis, genetic analysis, and molecular mechanisms. Prog Retin Eye Res.

[12] Yang HS, Lee JB, Yoon YH, Lee JY (2014). Correlation between spectral-domain OCT findings and visual acuity in X-linked retinoschisis. Invest Ophthalmol Vis Sci.

[13] Mann M, Hendrickson RC, Pandey A (2001). Analysis of proteins and proteomes by mass spectrometry. Annu Rev Biochem.

[14] Sugioka K, Saito A, Kusaka S, Kuniyoshi K, Shimomura Y (2017). Identification of vitreous proteins in retinopathy of prematurity. Biochem Biophys Res Commun.

[15] Sobrin L, Berrocal AM, Murray TG (2003). Retinal detachment 7 years after prophylactic schisis cavity excision in juvenile X-linked retinoschisis. Ophthalmic Surg Lasers Imaging.

[16] Wu WC, Drenser KA, Capone A, Williams GA, Trese MT (2007). Plasmin enzyme-assisted vitreoretinal surgery in congenital X-linked retinoschisis: surgical techniques based on a new classification system. Retina..

[17] Zeng Y, Takada Y, Kjellstrom S, Hiriyanna K, Tanikawa A, Wawrousek E (2004). RS-1 gene delivery to an adult Rs1h knockout mouse model restores ERG b-wave with reversal of the electronegative waveform of X-linked retinoschisis. Invest Ophthalmol Vis Sci.

[18] Min SH, Molday LL, Seeliger MW, Dinculescu A, Timmers AM, Janssen A (2005). Prolonged recovery of retinal structure/function after gene therapy in an Rs1h-deficient mouse model of X-linked juvenile retinoschisis. Mol Ther.

[19] Ou J, Vijayasarathy C, Ziccardi L, Chen S, Zeng Y, Marangoni D (2015). Synaptic pathology and therapeutic repair in adult retinoschisis mouse by AAV-RS1 transfer. J Clin Invest.

[20] Reid SN, Farber DB (2005). Glial transcytosis of a photoreceptor-secreted signaling protein, retinoschisin. Glia..

[21] Molday LL, Wu WW, Molday RS (2007). Retinoschisin (RS1), the protein encoded by the X-linked retinoschisis gene, is anchored to the surface of retinal photoreceptor and bipolar cells through its interactions with a Na/K ATPase-SARM1 complex. J Biol Chem.

[22] Joshi MM, Drenser K, Hartzer M, Dailey W, Capone A, Trese MT (2006). Intraschisis cavity fluid composition in congenital X-linked retinoschisis. Retina..

[23] Chiquet-Ehrismann R, Mackie EJ, Pearson CA, Sakakura T (1986). Tenascin: an extracellular matrix protein involved in tissue interactions during fetal development and oncogenesis. Cell..

[24] Chiquet-Ehrismann R, Chiquet M (2003). Tenascins: regulation and putative functions during pathological stress. J Pathol.

[25] Adler AJ, Severin KM (1981). Proteins of the bovine interphotoreceptor matrix: tissues of origin. Exp Eye Res.

[26] Gonzalez-Fernandez F (2003). Interphotoreceptor retinoid-binding protein--an old gene for new eyes. Vis Res.

[27] Parker RO, Crouch RK (2010). The interphotoreceptor retinoid binding (IRBP) is essential for normal retinoid processing in cone photoreceptors. Adv Exp Med Biol.

[28] Jin M, Li S, Nusinowitz S, Lloyd M, Hu J, Radu RA (2009). The role of interphotoreceptor retinoid-binding protein on the translocation of visual retinoids and function of cone photoreceptors. J Neurosci.

[29] Sato K, Li S, Gordon WC, He J, Liou GI, Hill JM (2013). Receptor interacting protein kinase-mediated necrosis contributes to cone and rod photoreceptor degeneration in the retina lacking interphotoreceptor retinoid-binding protein. J Neurosci.

[30] Miyake Y, Shiroyama N, Ota I, Horiguchi M (1993). Focal macular electroretinogram in X-linked congenital retinoschisis. Invest Ophthalmol Vis Sci.

[31] Peachey NS, Fishman GA, Derlacki DJ, Brigell MG (1987). Psychophysical and electroretinographic findings in X-linked juvenile retinoschisis. Arch Ophthalmol.

[32] Caspi RR, Chan CC, Wiggert B, Chader GJ (1990). The mouse as a model of experimental autoimmune uveoretinitis (EAU). Curr Eye Res.

[33] Solomon A, Banin E, Anteby I, Benezra D (1999). Retinitis pigmentosa, coats disease and uveitis. Eur J Ophthalmol.

[34] Morohoshi K, Ohbayashi M, Patel N, Chong V, Bird AC, Ono SJ (2012). Identification of anti-retinal antibodies in patients with age-related macular degeneration. Exp Mol Pathol.

